# Semaglutide-Induced Atypical Pustular Drug Eruption: A Case Report

**DOI:** 10.7759/cureus.101016

**Published:** 2026-01-07

**Authors:** Destinee C Elliott, Francesca L Veon, Jeffrey D McBride, Jarad Levin

**Affiliations:** 1 Department of Dermatology, University of Oklahoma Health Sciences Center, Oklahoma City, USA; 2 Mark Allen Everett Department of Dermatology, University of Oklahoma Health Campus, Oklahoma City, USA

**Keywords:** acute generalized exanthematous pustulosis (agep), cutaneous drug eruption, propylene glycol, semaglutide glp-1 analogue, type iv hypersensitivity reaction, types 2 diabetes

## Abstract

Glucagon-like peptide 1 (GLP-1) agonists continue to gain popularity in treating an increasing number of chronic conditions. As such, it is imperative that providers keep a vigilant eye on patients utilizing these medicines, monitoring for both known and novel side effects. Herein, we present the case of a patient who developed a rash with features characteristic of acute generalized exanthematous pustulosis (AGEP) one month after increasing her dose of semaglutide (Ozempic®). Her eruption occurred outside of the normal timeline for AGEP but had phenotypic and histopathologic findings consistent with this condition, emphasizing the need for providers to maintain a broad differential when assessing cutaneous eruptions. In addition, this case represents the need to do a thorough ingredient analysis when investigating new symptoms, as it was elucidated that her reaction was likely due to the excipient propylene glycol rather than the active ingredient in semaglutide (Ozempic®). The safety of patients using semaglutide requires close correlation of clinical presentation, histopathologic findings, and multidisciplinary collaboration with pharmacists.

## Introduction

Cutaneous drug eruptions are common adverse effects of pharmacologic therapies, with clinical and histopathologic findings varying widely. Accurate classification requires close correlation of these elements. Herein, we report the case of a 43-year-old female who developed an atypical pustular eruption with features mimicking an acute generalized exanthematous pustulosis (AGEP), likely triggered by semaglutide (Ozempic®), a glucagon-like peptide-1 (GLP-1) receptor agonist. AGEP is a type IV hypersensitivity reaction mediated by CXCL8/IL-8-producing T-cells, leading to neutrophilic activation and infiltration [1]. The classic presentation of AGEP is characterized by multiple small, non-follicular, sterile pustules on an erythematous base that typically originate on the face and subsequently spread over the body. Systemic symptoms can include generalized itch, a burning sensation, and fever. Over 90% of the reported cases of AGEP are attributed to drug reactions, but infectious causes have also been described [2]. Onset is typically within 24-48 hours of starting the drug, with resolution usually occurring two weeks after stopping the offending agent [3]; rarely, the reaction can be widespread and fatal due to renal or hepatic failure [2]. Among the wide variety of pharmaceuticals linked to AGEP, antibiotics stand out as the most frequently cited agents. Conversely, the most frequently reported adverse effects of semaglutide (Ozempic®) are generally gastrointestinal, but various cutaneous effects are being increasingly recognized. Despite the growing number of dermatologic conditions being discovered, cases of AGEP-like reactions remain rare [4]. This case highlights the need for a broad differential when evaluating cutaneous eruptions occurring in patients on novel or increasingly used medications.

## Case presentation

A 43-year-old female with type II diabetes mellitus, hypertension, hyperlipidemia, and recurrent genital herpes presented with the abrupt onset of a diffuse, pruritic, burning rash. She was treated at an outside emergency department two days earlier for chills, itching, and a rash initially affecting her right arm and torso. She received cephalexin, meloxicam, and diphenhydramine without relief. The rash progressed to her flexures, chest, trunk, and legs. Vesiculopustular lesions developed on her face and postauricular areas. Prednisone and hydroxyzine were initiated, and she presented to our clinic the next day.

She denied constitutional symptoms as well as insect or other environmental exposures. After a thorough medication reconciliation, it was found that her semaglutide (Ozempic®) dose was increased from 0.5 mg to 1 mg approximately 30 days prior. There were no other changes to her chronic medications, including valacyclovir, an antihypertensive, and a statin, increasing the likelihood that the reaction was due to semaglutide (Ozempic®).

**Figure 1:**
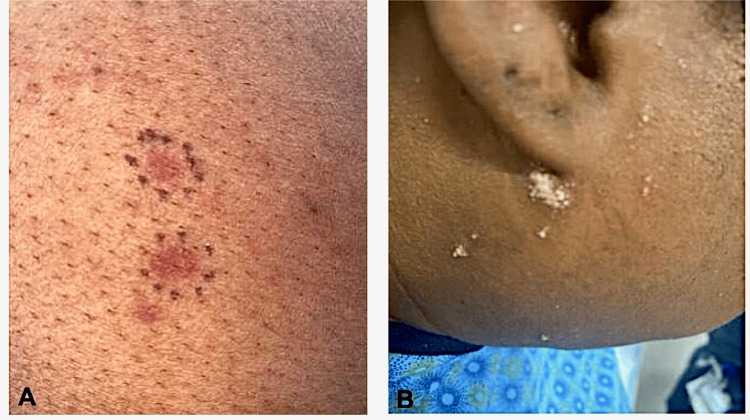
Violaceous-erythematous macules and vesiculopustular lesions A: Violaceous-erythematous macules located on the patient's lower extremity B: Grouped vesiculopustular lesions located on the patient's mandible

Physical exam revealed erythematous to violaceous macules and patches on the bilateral flexures, trunk, and legs without palm or sole involvement (Figure 1A). Fading macules most consistent with post-inflammatory hyperpigmentation were seen elsewhere. Grouped vesiculopustular lesions were present on the face and postauricular areas (Figure 1B). Preliminary differential diagnoses aside from drug eruption included HSV, vasculitis, bacterial/fungal infection, pustular psoriasis, and neoplastic etiologies. Her infectious workup, including cultures of the lesions and a sample to be used for PCR to assess HSV status, was obtained and was non-reactive. Punch biopsies of the postauricular region and the thigh were submitted for H&E and direct immunofluorescence (DIF). Prednisone was stopped; cetirizine and hydroxyzine were continued.

**Figure 2:**
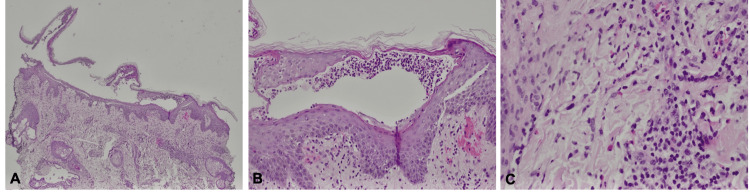
Histological findings A: 40X total magnification punch biopsy of pustular drug eruption with features of AGEP B: 100X total magnification showing subcorneal and intraepidermal neutrophilic inclusions C: 200X total magnification showing perivascular and interstitial lymphohistiocytic inflammation with intraluminal eosinophils and neutrophils AGEP - acute generalized exanthematous pustulosis

Additional cultures and DIF were unremarkable. H&E from the postauricular lesion showed intraepidermal and subcorneal neutrophils with spongiosis, and superficial perivascular lymphohistiocytic infiltrates, neutrophils, and eosinophils in the dermis (Figure 2). The thigh biopsy revealed similar findings, most consistent with an atypical AGEP-like eruption. These histological findings decreased the likelihood of the other differential diagnoses.

Thereafter, semaglutide (Ozempic®) was discontinued, and an alternative glycemic therapy was discussed with her primary care provider. Tacrolimus was prescribed for facial lesions, and triamcinolone cream for symptomatic pruritus. At one-month follow-up, the eruption had resolved with residual facial hyperpigmentation, further supporting the GLP-1 agonist as the likely trigger of her hypersensitivity reaction. The patient was counseled to avoid future semaglutide (Ozempic®) use due to recurrence risk.

An ingredient analysis conducted by a pharmacy colleague comparing semaglutide (Ozempic®) with previously tolerated insulin aspart (Novolog®) revealed propylene glycol, present in semaglutide (Ozempic®) but not insulin aspart (Novolog®), as the only differing excipient. This supported semaglutide (Ozempic®) as the most probable culprit. A trial of tirzepatide, which lacks propylene glycol, was recommended as a safer alternative.

## Discussion

Obesity carries significant physical and psychological burdens. A Global Burden of Disease study identified metabolic disease as a significant risk factor for global mortality [5], increasing the urgency for effective weight-loss medications. Semaglutide (Ozempic®), a GLP-1 receptor agonist, mimics incretin hormones to increase satiety and insulin secretion [6]. Initially approved for type 2 diabetes, it is now widely used for weight loss with substantial success.

As GLP-1 agonist use rises, so do reports of adverse events. Gastrointestinal symptoms remain the most common [4], but cutaneous reactions are becoming increasingly recognized. These include injection-site reactions, telogen effluvium, urticaria, bullous pemphigoid, fixed drug eruptions, and other delayed hypersensitivity responses [4,7]. Despite the increase in dermatologic conditions resulting from GLP-1 agonist use, few reports of AGEP-like reactions exist in the literature, highlighting our case's novelty. Another case report introduces an AGEP-like reaction following semaglutide injection that did not present with pustules [8], further emphasizing the importance of maintaining a broad differential when investigating cutaneous eruptions in patients taking these medications, as they could present differently than the classical clinical picture.

Histopathology in our patient showed hallmark AGEP features: subcorneal and intraepidermal pustules, spongiosis, and perivascular lymphohistiocytic infiltrates with eosinophils. Clinically, she exhibited widespread erythematous macules, flexural involvement, and sterile vesiculopustules, features consistent with an AGEP-like pustular drug eruption. However, the one-month latency was atypical, as true AGEP typically appears within days [9]. The concomitant medications, cephalexin and meloxicam, were initially considered as potential triggers for the AGEP-like reaction, as reports of both medications causing the eruption have been described [10,11]. Cephalexin, as a beta-lactam antibiotic, received increased attention, as this class of drugs is the most common in triggering AGEP [10]. However, the temporal association of the patient's reaction with the increase in semaglutide (Ozempic®) dose, not the subsequent medications used to treat the symptoms, and resolution of rash soon after discontinuation, increases the likelihood that the GLP-1 agonist was responsible. This highlights the need for clinicopathologic correlation in cases of delayed or evolving hypersensitivity. Objectively, the patient's reaction scored a four on the Naranjo scale, indicating that the AGEP-like reaction was possibly due to semaglutide (Ozempic®). However, no confirmatory laboratory or patch testing was completed, which could have strengthened this idea. Future encounters of such a reaction should be investigated through these mechanisms to increase confidence in the diagnosis. 

Another key consideration in adverse reactions is excipient sensitivity. In this case, propylene glycol was the only excipient present in semaglutide (Ozempic®) but absent in previously tolerated insulin aspart (Novolog®). Propylene glycol, a common vehicle used in medications, is a known sensitizer associated with eczematous and systemic eruptions [12]. Its role here supports the idea that reactions may stem not just from the active drug, but from a co-reaction with the inactive components of pharmaceuticals. Recognizing such triggers allows for informed substitution, such as recommending tirzepatide, which lacks propylene glycol.

## Conclusions

As semaglutide and similar agents become more widely used, clinicians must remain vigilant for cutaneous side effects. Our case emphasizes that these pharmaceuticals are capable of producing reactions that are quite rare, and providers should continue to report them in the literature as they occur. This practice will assist in the continuous development of evidence-based guidelines for prescribing these medications. Additionally, our case underscores the importance of collaboration with pharmacists as a part of the multidisciplinary care team. Eliciting their expertise led to the prompt identification of the likely causative agent of our patient's reaction and the introduction of a safer alternative. In conclusion, early recognition, clinicopathologic correlation, and attention to both active ingredients and excipients are essential for diagnosis and safe patient care.

## References

[1] Chu MT, Chang WC, Pao SC, Hung SI (2023). Delayed drug hypersensitivity reactions: molecular recognition, genetic susceptibility, and immune mediators. Biomedicines.

[2] Stadler PC, Oschmann A, Kerl-French K, Maul JT, Oppel EM, Meier-Schiesser B, French LE (2023). Acute generalized exanthematous pustulosis: clinical characteristics, pathogenesis, and management. Dermatology.

[3] Szatkowski J, Schwartz RA (2015). Acute generalized exanthematous pustulosis (AGEP): a review and update. J Am Acad Dermatol.

[4] Burke OM, Sa B, Cespedes DA, Tosti A (2025). Dermatologic implications of glucagon-like peptide-1 receptor agonist medications. Skin Appendage Disord.

[5] Zhang H, Zhou XD, Shapiro MD (2024). Global burden of metabolic diseases, 1990-2021. Metabolism.

[6] Moore PW, Malone K, VanValkenburg D (2023). GLP-1 agonists for weight loss: pharmacology and clinical implications. Adv Ther.

[7] Talasila S, Waseh S, Liu JY, Khalifeh I, Metkowski AR, Hsu S (2025). Semaglutide-induced fixed drug eruption. JAAD Case Rep.

[8] Shetty NP, Veenstra J (2024). Acute generalized exanthematous pustulosis sine pustules following semaglutide injection. JAAD Case Rep.

[9] Sidoroff A, Dunant A, Viboud C (2007). Risk factors for acute generalized exanthematous pustulosis (AGEP)-results of a multinational case-control study (EuroSCAR). Br J Dermatol.

[10] Lei H, Deng H, Liu X, Li Z, Wang C (2022). Clinical features, diagnosis and management of cephalosporin-induced acute generalized exanthematous pustulosis. J Clin Pharm Ther.

[11] Vallejo-Yagüe E, Martinez-De la Torre A, Mohamad OS, Sabu S, Burden AM (2022). Drug triggers and clinic of acute generalized exanthematous pustulosis (AGEP): a literature case series of 297 patients. J Clin Med.

[12] Pemberton MA, Kimber I (2023). Propylene glycol, skin sensitisation and allergic contact dermatitis: a scientific and regulatory conundrum. Regul Toxicol Pharmacol.

